# Supplementary material for: Gene signatures associated with barrier dysfunction and infection in oral lichen planus identified by analysis of transcriptomic data
Source: PLoS One. 2021 Sep 10;16(9):e0257356. doi: 10.1371/journal.pone.0257356 (PMC8432868; doi:10.1371/journal.pone.0257356)
Supplement: S1 Table — (PDF) [file pone.0257356.s001.pdf]

S1 Table. Differentially expressed genes (DEGs) in the epithelium whole dataset

Gene symbol	Fold-change	*p*-value	*q*-value
LOR	39.08	6.2E-04	0.0200
SPRR2G	30.22	7.6E-05	0.0105
LCE3D	21.98	5.5E-05	0.0097
KRT17	21.48	2.7E-05	0.0088
S100A7	19.96	4.5E-05	0.0095
LCE3E	18.42	9.8E-05	0.0113
ASPRV1	17.66	2.9E-03	0.0346
RPTN	16.54	9.6E-04	0.0228
LCE3A	12.66	8.0E-04	0.0217
FLG	10.73	1.1E-03	0.0242
ALOX12B	10.57	4.9E-05	0.0096
KPRP	10.14	1.7E-03	0.0276
KRT16	9.86	8.1E-05	0.0088
TMEM45A	9.39	7.4E-04	0.0313
SPRR2B	8.49	1.8E-04	0.0129
PI3	7.88	4.7E-04	0.0181
KRT16P2	7.48	4.7E-05	0.0189
S100P	7.39	1.4E-03	0.0262
DEFB103A	7.32	1.0E-03	0.0281
C6ORF15	7.27	5.5E-03	0.0453
SPRR2C	7.00	2.0E-03	0.0294
SPRR2E	6.83	5.4E-03	0.0448
IL36G	6.72	1.3E-03	0.0257
DEFB4A	6.72	6.7E-03	0.0494
RNASE7	6.23	1.8E-03	0.0281
IL36RN	6.19	1.1E-04	0.0118
SPINK7	6.13	4.4E-03	0.0411
IFI27	5.97	2.9E-03	0.0346
DMKN	5.70	3.5E-05	0.0089
KRT10	5.48	2.2E-04	0.0136
LY6G6C	5.07	9.5E-05	0.0112
WFDC5	4.97	8.8E-04	0.0459
SERPINB7	4.76	1.3E-03	0.0253
KRT80	4.74	1.2E-03	0.0246
PLA2G4D	4.64	1.9E-03	0.0288
KLK7	4.57	6.7E-03	0.0494
LCN2	4.43	5.3E-03	0.0448
DEFB103B	4.41	4.7E-04	0.0181
IGFL2	4.29	6.8E-03	0.0500
CALML5	4.24	6.0E-03	0.0471
KLK12	4.16	1.5E-03	0.0176
AAK1	4.14	1.1E-03	0.0243
ADAP2	4.13	5.9E-05	0.0100
IL1B	3.96	8.1E-05	0.0105
RDH12	3.95	5.0E-06	0.0069
IL1F10	3.91	2.8E-03	0.0342
SIAE	3.86	5.8E-03	0.0462
KLK10	3.81	4.5E-05	0.0095
DUSP14	3.79	1.3E-04	0.0122
WDR66	3.75	5.9E-03	0.0469
ALOXE3	3.70	1.6E-03	0.0270
S100A12	3.67	2.7E-03	0.0337
PNLIPRP3	3.62	5.1E-04	0.0187

LRRC20	3.62	1.2E-03	0.0245
SH3GL3	3.57	5.7E-04	0.0191
TUBB3	3.46	5.4E-04	0.0189
PRSS3	3.42	1.6E-03	0.0270
CCND2	3.38	7.1E-04	0.0208
SUGCT	3.29	5.6E-03	0.0456
SPRR1B	3.26	3.6E-03	0.0375
RFTN1	3.26	1.4E-04	0.0125
PSG4	3.25	5.5E-03	0.0453
FETUB	3.12	6.3E-03	0.0481
RGS20	3.02	1.8E-04	0.0129
FAM89A	3.01	4.9E-04	0.0233
C10ORF99	2.95	4.9E-03	0.0431
LYPD5	2.95	6.3E-03	0.0483
FEZ1	2.89	4.5E-03	0.0414
BNIP3	2.87	6.3E-04	0.0200
ERP27	2.85	1.6E-03	0.0269
CA2	2.84	2.8E-04	0.0189
CD36	2.80	2.0E-03	0.0412
COL3A1	2.79	5.5E-03	0.0454
COL4A1	2.76	1.3E-03	0.0256
CTSL	2.75	4.6E-03	0.0417
FLRT3	2.72	3.8E-03	0.0386
RIMS3	2.62	1.4E-03	0.0260
ATP10B	2.60	3.1E-06	0.0069
BAMBI	2.57	6.3E-03	0.0483
COL6A3	2.56	9.7E-04	0.0236
FAM25G	2.55	1.9E-03	0.0290
NIPAL4	2.54	7.1E-04	0.0208
HIGD1A	2.53	3.7E-04	0.0271
GJB6	2.50	3.6E-03	0.0378
FAM25C	2.50	3.8E-03	0.0387
SLC31A2	2.49	9.7E-04	0.0230
TNC	2.49	5.0E-03	0.0436
KLK5	2.47	4.1E-03	0.0235
GJA1	2.45	3.0E-05	0.0088
QSOX1	2.42	6.3E-04	0.0200
HIST1H2BD	2.42	1.0E-03	0.0234
ABCG4	2.41	3.0E-03	0.0351
CYP4F22	2.40	1.2E-04	0.0122
EML1	2.39	5.3E-04	0.0189
FAT1	2.38	2.3E-03	0.0189
CPE	2.38	2.9E-03	0.0346
ISG20	2.37	4.1E-03	0.0404
FLVCR2	2.36	3.3E-05	0.0089
ATP6V1C2	2.36	6.2E-03	0.0479
SLC23A1	2.36	7.3E-04	0.0208
SLC5A1	2.36	3.0E-03	0.0351
AVPI1	2.34	2.5E-03	0.0323
LUM	2.31	3.4E-03	0.0369
DFNA5	2.30	2.9E-03	0.0345
DSG1	2.29	2.2E-03	0.0310
PPP1R14C	2.28	2.3E-05	0.0088
COL5A2	2.28	4.7E-03	0.0427
BTG3	2.27	3.8E-05	0.0090
HTRA1	2.25	1.2E-04	0.0122

SERPINE2	2.24	6.6E-03	0.0490
STK40	2.24	1.3E-03	0.0254
KLK8	2.22	1.9E-03	0.0288
PRSS23	2.22	3.4E-03	0.0370
RCAN1	2.18	3.6E-03	0.0378
NABP1	2.18	3.7E-03	0.0383
LGALSL	2.18	1.8E-03	0.0281
ERRFI1	2.16	4.0E-04	0.0172
HYAL1	2.16	4.2E-03	0.0406
PDGFRB	2.15	4.8E-03	0.0430
GGH	2.15	1.1E-03	0.0241
PTPRZ1	2.14	1.1E-03	0.0241
ELOVL4	2.12	6.5E-03	0.0487
UBR4	2.10	4.6E-03	0.0419
AKR1B15	2.09	4.4E-03	0.0411
COL1A2	2.09	9.5E-03	0.0431
CRISPLD2	2.08	4.3E-03	0.0408
PRRX2	2.07	3.5E-03	0.0375
CEMIP	2.07	1.8E-04	0.0129
UNC93A	2.07	8.3E-04	0.0218
PLCXD1	2.04	6.0E-03	0.0474
DUSP6	2.03	5.7E-03	0.0460
SLC16A9	2.03	5.7E-04	0.0191
DOCK11	2.03	5.1E-06	0.0069
MLLT11	2.02	2.4E-04	0.0140
MOCOS	2.00	1.7E-04	0.0127
SPIRE1	2.00	8.3E-04	0.0218
MYO5A	2.00	8.9E-04	0.0223
ZBTB42	-2.00	5.4E-05	0.0097
FRAS1	-2.00	4.0E-03	0.0397
SYT15	-2.02	1.5E-03	0.0265
HIST1H4C	-2.03	7.2E-04	0.0208
SHMT1	-2.04	2.2E-03	0.0304
PTN	-2.04	1.2E-05	0.0083
PPARGC1A	-2.04	1.1E-04	0.0118
ZNF395	-2.06	2.2E-03	0.0309
SF3B3	-2.06	1.9E-05	0.0083
FAM189A2	-2.08	1.9E-03	0.0289
PAFAH1B3	-2.09	3.1E-03	0.0355
MIR99AHG	-2.10	3.2E-03	0.0363
ITPRIPL2	-2.11	1.7E-04	0.0128
THEM6	-2.15	1.1E-04	0.0118
NFIX	-2.15	3.5E-03	0.0375
CYP11A1	-2.15	5.5E-04	0.0189
STOX2	-2.18	9.4E-04	0.0227
OPLAH	-2.19	1.0E-03	0.0233
AIM1L	-2.23	2.1E-04	0.0142
CCND1	-2.24	1.3E-04	0.0122
ZDHHC11	-2.25	3.2E-03	0.0359
ROR1	-2.25	1.8E-03	0.0281
SLC25A23	-2.28	4.2E-03	0.0406
TGFBR3	-2.31	3.4E-03	0.0374
NFIB	-2.31	2.6E-03	0.0332
AMER1	-2.35	1.3E-05	0.0083
IGFBP3	-2.37	1.8E-03	0.0284
RGMA	-2.44	1.6E-05	0.0083

ZBTB7C	-2.45	7.3E-04	0.0208
CUEDC1	-2.48	2.6E-03	0.0333
AKR1A1	-2.48	4.5E-04	0.0179
FGFR3	-2.49	1.6E-03	0.0269
ASS1	-2.51	4.3E-03	0.0408
KRT31	-2.53	9.2E-04	0.0227
MAOB	-2.57	1.1E-04	0.0118
ALDH3A2	-2.58	2.1E-04	0.0105
ZNF296	-2.60	7.5E-04	0.0210
ANKRD20A1	-2.62	4.1E-04	0.0174
MAOA	-2.65	3.3E-03	0.0368
SCIN	-2.66	3.7E-05	0.0089
CYP4F12	-2.66	1.3E-03	0.0255
PGD	-2.67	5.9E-03	0.0470
PAQR8	-2.70	2.5E-04	0.0141
CLDN23	-2.71	2.7E-05	0.0088
GPD1L	-2.77	1.4E-04	0.0125
PHGDH	-2.81	2.2E-03	0.0310
KRT8	-2.87	5.2E-05	0.0096
BCL11A	-2.90	1.0E-04	0.0115
HMGCS1	-2.96	6.0E-06	0.0069
TM4SF1	-2.98	2.7E-03	0.0337
TF	-3.06	3.5E-03	0.0375
E2F2	-3.07	1.2E-04	0.0121
RHCG	-3.33	5.6E-03	0.0456
RBM20	-3.40	1.0E-03	0.0233
WNK4	-3.63	2.4E-03	0.0321
COX7A1	-3.66	1.6E-06	0.0069
CLDN7	-3.83	5.6E-04	0.0191
CYP3A5	-4.00	2.9E-04	0.0148
ETNK2	-4.01	7.6E-05	0.0105
MAMDC2	-4.15	1.7E-03	0.0279
MT1G	-4.26	6.5E-03	0.0487
MUC21	-6.96	6.3E-03	0.0396
CES1	-6.97	2.9E-03	0.0349
