# Supplementary material for: Gene signatures associated with barrier dysfunction and infection in oral lichen planus identified by analysis of transcriptomic data
Source: PLoS One. 2021 Sep 10;16(9):e0257356. doi: 10.1371/journal.pone.0257356 (PMC8432868; doi:10.1371/journal.pone.0257356)
Supplement: S2 Table — (PDF) [file pone.0257356.s002.pdf]

S2 Table. Differentially expressed genes (DEGs) in the epithelium partial dataset

Gene symbol	Fold-change	*p*-value	*q*-value
SPRR2G	80.66	1.7E-07	0.0002
LOR	75.04	1.3E-05	0.0019
LCE3D	54.39	1.2E-05	0.0019
ASPRV1	50.03	2.3E-03	0.0182
RPTN	45.16	5.0E-08	0.0001
S100A7	41.53	1.4E-03	0.0139
LCE3E	37.64	1.7E-07	0.0002
KRT17	36.74	2.1E-04	0.0054
LCE3A	28.04	3.1E-05	0.0025
KPRP	25.90	9.4E-06	0.0017
ALOX12B	20.06	4.2E-06	0.0011
TMEM45A	18.65	1.1E-04	0.0072
DEFB4A	17.65	5.9E-03	0.0317
FLG	16.49	2.3E-03	0.0181
KRT16	15.69	6.7E-06	0.0014
S100P	15.62	7.1E-04	0.0098
C6ORF15	15.12	1.2E-02	0.0479
IL36G	14.91	1.1E-06	0.0005
DEFB103A	12.88	8.7E-04	0.0141
SPRR2B	12.46	4.7E-04	0.0079
RNASE7	12.39	2.5E-04	0.0059
SPRR2E	11.98	6.1E-03	0.0325
PI3	11.61	1.3E-03	0.0133
SPRR2C	11.34	3.9E-04	0.0072
KRT16P2	11.12	7.1E-07	0.0080
IL36RN	10.81	1.4E-04	0.0045
SPINK7	10.19	4.2E-03	0.0257
ARG1	10.10	2.5E-03	0.0190
DMKN	9.67	6.4E-06	0.0044
KRT80	8.72	1.0E-04	0.0040
WFDC5	8.62	1.4E-05	0.0101
SPRR2D	8.11	9.7E-03	0.0428
IGFL2	7.93	2.2E-04	0.0056
SERPINB7	7.91	1.1E-05	0.0018
KRT10	7.84	4.2E-03	0.0256
LCN2	7.65	1.4E-03	0.0141
LY6G6C	7.48	1.7E-05	0.0020
CALML5	7.27	3.8E-03	0.0242
S100A12	7.11	4.7E-04	0.0079
KLK7	7.09	1.6E-03	0.0147
PLA2G4D	6.88	9.0E-03	0.0407
IL1F10	6.84	1.9E-03	0.0163
DEFB103B	6.80	5.9E-03	0.0318
WDR66	6.76	3.0E-03	0.0211
AAK1	6.55	1.1E-04	0.0041
SIAE	6.18	1.2E-03	0.0130
ADAP2	6.17	2.1E-08	0.0001
ALOXE3	6.04	7.5E-05	0.0035
PSG4	5.75	4.4E-03	0.0263
FETUB	5.67	2.4E-04	0.0058
PRSS3	5.60	1.6E-04	0.0047
DUSP14	5.57	4.1E-05	0.0028
PNLIPRP3	5.57	6.2E-04	0.0091

SLURP1	5.52	1.6E-03	0.0149
KLK10	5.42	1.5E-05	0.0019
LYPD5	5.40	7.1E-05	0.0034
KLK12	5.33	1.0E-03	0.0040
TUBB3	5.24	3.5E-05	0.0027
LRRC20	5.24	4.7E-03	0.0263
IL1B	5.09	2.3E-04	0.0057
SPRR1B	5.07	3.0E-04	0.0064
ANXA9	4.71	7.1E-03	0.0353
COL1A2	4.70	7.7E-03	0.0372
RDH12	4.68	5.2E-05	0.0029
ERP27	4.68	5.7E-06	0.0014
RGS20	4.55	1.9E-05	0.0021
RFTN1	4.55	3.7E-04	0.0070
FAM89A	4.52	3.9E-05	0.0099
RBP1	4.41	2.3E-03	0.0182
CCND2	4.39	8.7E-09	0.0001
BNIP3	4.38	3.6E-05	0.0027
CTSV	4.35	2.5E-03	0.0189
KLK5	4.24	4.0E-06	0.0029
FEZ1	4.20	4.2E-03	0.0257
FLRT3	4.15	9.7E-04	0.0116
ODC1	4.05	3.1E-03	0.0216
CA2	4.00	1.1E-05	0.0012
C10ORF99	3.94	2.2E-03	0.0178
LYNX1	3.90	2.1E-04	0.0055
YOD1	3.90	4.1E-04	0.0073
CDH3	3.89	4.9E-03	0.0282
COL3A1	3.84	6.5E-03	0.0337
NIPAL4	3.78	1.6E-05	0.0019
CTSL	3.75	1.8E-03	0.0166
SLC31A2	3.75	4.6E-05	0.0029
COL4A1	3.72	5.0E-03	0.0283
ISG20	3.69	4.5E-04	0.0077
TNC	3.64	1.2E-04	0.0042
RNF39	3.63	2.5E-04	0.0059
SH3GL3	3.58	3.1E-03	0.0215
ATP6V1C2	3.57	5.8E-04	0.0089
FAM25C	3.55	2.0E-03	0.0166
HIGD1A	3.47	6.5E-05	0.0033
FAM25G	3.46	2.0E-03	0.0166
HIST1H2BD	3.44	3.6E-05	0.0027
ABCG4	3.42	5.1E-03	0.0288
LUM	3.40	1.9E-05	0.0021
GJB6	3.39	7.8E-04	0.0103
COL6A3	3.38	2.8E-04	0.0063
RIMS3	3.29	2.0E-03	0.0166
EML1	3.24	7.4E-05	0.0035
HMOX1	3.22	1.3E-03	0.0135
KLK8	3.19	2.8E-04	0.0062
EFR3B	3.19	8.3E-03	0.0388
SLC5A1	3.15	4.5E-03	0.0265
AVPI1	3.14	2.2E-04	0.0056
TCEAL9	3.13	8.5E-06	0.0062
TGFBI	3.12	8.4E-03	0.0389
LGALSL	3.10	1.5E-05	0.0019

SLC23A1	3.06	4.4E-06	0.0011
THY1	3.05	1.0E-02	0.0446
FAT1	3.04	4.3E-03	0.0146
KLK13	3.03	1.2E-02	0.0483
QSOX1	2.99	6.9E-04	0.0096
DFNA5	2.95	2.7E-03	0.0197
DSG1	2.93	3.4E-04	0.0069
RGS2	2.92	5.8E-04	0.0089
CPE	2.89	5.6E-04	0.0086
FLVCR2	2.88	4.6E-04	0.0078
ERRFI1	2.86	4.9E-05	0.0029
CYP4F22	2.85	6.5E-04	0.0094
UBR4	2.85	8.2E-06	0.0015
STK40	2.82	4.6E-05	0.0029
SAMD9	2.80	6.7E-04	0.0095
GJA1	2.79	1.1E-04	0.0040
IL1A	2.79	8.3E-03	0.0388
ATP10B	2.78	4.5E-04	0.0077
RCAN1	2.77	4.8E-03	0.0276
BEX3	2.70	5.7E-07	0.0007
ELOVL4	2.70	5.4E-04	0.0085
PLCXD1	2.69	4.0E-04	0.0073
CRISPLD2	2.69	2.7E-03	0.0198
FBLN2	2.68	6.0E-03	0.0319
MAST4	2.68	3.5E-03	0.0230
LPXN	2.67	1.8E-03	0.0157
CNTNAP2	2.67	4.1E-03	0.0255
PAM	2.66	1.1E-03	0.0120
FBN2	2.66	9.2E-03	0.0414
BTG3	2.65	3.6E-03	0.0043
EPB41L3	2.64	1.2E-03	0.0126
PRSS23	2.64	6.6E-03	0.0337
LAMA3	2.64	1.4E-02	0.0432
TP53INP2	2.61	4.3E-03	0.0259
TUBB2A	2.61	8.8E-03	0.0404
SPIRE1	2.60	4.7E-04	0.0079
NDEL1	2.60	7.9E-05	0.0036
CPA3	2.60	1.4E-03	0.0139
SPRR2A	2.58	6.1E-03	0.0325
PPP1R14C	2.56	2.1E-03	0.0169
UNC93A	2.56	3.9E-03	0.0247
PTPRZ1	2.55	2.6E-03	0.0196
PRKCDBP	2.54	9.8E-03	0.0430
NABP1	2.53	6.4E-03	0.0333
PLBD1	2.52	5.9E-03	0.0317
SMOX	2.52	9.1E-03	0.0411
SERPINB8	2.51	1.6E-05	0.0056
TGM5	2.50	8.2E-03	0.0386
LIMA1	2.49	2.1E-04	0.0054
RAB31	2.48	3.5E-04	0.0069
CEMIP	2.48	1.9E-04	0.0051
MAP2	2.47	4.0E-06	0.0011
MYO1B	2.47	1.1E-03	0.0190
DNAJB6	2.47	2.7E-05	0.0025
MYO5A	2.47	2.4E-03	0.0183
TYMP	2.47	5.1E-03	0.0289

MLLT11	2.45	8.5E-05	0.0037
TGFA	2.43	2.9E-04	0.0064
INA	2.43	7.8E-03	0.0374
MTHFD1L	2.42	3.2E-09	0.0000
CWH43	2.41	2.9E-04	0.0064
SLC39A2	2.41	6.7E-04	0.0095
DUSP6	2.40	1.1E-02	0.0469
SLC39A6	2.40	2.9E-04	0.0235
IFFO2	2.40	8.4E-03	0.0391
ADGRL2	2.39	2.8E-05	0.0025
CD207	2.39	5.8E-05	0.0032
LITAF	2.36	7.9E-04	0.0104
HTRA1	2.36	1.9E-04	0.0051
ABHD5	2.35	2.4E-03	0.0187
ARL5A	2.34	3.0E-03	0.0284
FKBP1A	2.34	1.5E-03	0.0273
TMPRSS11D	2.33	2.5E-03	0.0189
HOPX	2.33	3.9E-04	0.0072
PYCARD	2.32	3.2E-06	0.0011
STX11	2.32	3.4E-03	0.0229
RAB32	2.32	1.6E-03	0.0150
HPSE	2.31	9.7E-04	0.0084
SPRR2F	2.31	1.2E-02	0.0477
FSCN1	2.31	1.6E-04	0.0047
PDGFRB	2.30	9.0E-03	0.0407
FAM167A	2.29	4.7E-04	0.0079
TIGAR	2.29	3.7E-04	0.0071
LAMP5	2.28	4.8E-03	0.0277
CYP2C18	2.27	6.5E-03	0.0335
LGMN	2.27	2.2E-04	0.0056
THBS2	2.27	8.6E-03	0.0398
HIST2H2AC	2.26	3.4E-04	0.0069
MOCOS	2.25	2.4E-03	0.0183
PRNP	2.25	3.1E-04	0.0167
GPR137B	2.23	2.0E-03	0.0166
FAM43A	2.23	9.1E-03	0.0410
MAPK6	2.23	3.5E-04	0.0069
HIST1H2BC	2.23	1.2E-02	0.0479
COMP	2.22	8.3E-03	0.0388
KIF5B	2.19	1.2E-04	0.0042
SDCBP2	2.19	7.8E-03	0.0374
PRKCSH	2.18	3.6E-05	0.0027
DOCK11	2.18	2.3E-07	0.0002
PRRX2	2.18	1.7E-03	0.0153
RAB9A	2.18	8.5E-05	0.0037
NCCRP1	2.18	2.0E-03	0.0165
HIST2H2AA4	2.17	4.9E-05	0.0029
TTC39B	2.17	1.0E-03	0.0119
ULBP2	2.16	1.1E-02	0.0467
CST6	2.16	2.0E-03	0.0166
AFAP1L2	2.16	3.1E-04	0.0066
GABARAPL2	2.16	1.6E-04	0.0046
RAB38	2.15	1.9E-03	0.0161
HSPA8	2.15	2.2E-04	0.0372
F2RL1	2.15	3.5E-04	0.0125
CEBPB	2.15	3.1E-04	0.0065

ANTXR2	2.15	1.2E-02	0.0491
FAM69A	2.15	3.9E-05	0.0027
HIST2H2AA3	2.14	2.4E-04	0.0049
HCST	2.14	6.3E-02	0.0184
CD83	2.13	1.5E-02	0.0425
CASP4	2.13	1.8E-04	0.0050
GJB2	2.12	6.8E-04	0.0096
DLX5	2.11	4.4E-03	0.0264
SLC15A1	2.11	3.1E-03	0.0217
TMEM173	2.10	3.8E-03	0.0243
C12ORF56	2.10	2.4E-03	0.0184
TMEM54	2.09	1.3E-05	0.0019
H2AFJ	2.09	1.1E-03	0.0120
MAPRE2	2.08	3.8E-03	0.0242
MACF1	2.08	4.1E-06	0.0011
UBE2F	2.08	1.7E-05	0.0020
NRIP1	2.08	1.0E-05	0.0017
CHIC2	2.07	2.3E-03	0.0181
JMJD7-PLA2G4B	2.07	9.1E-04	0.0112
PRSS2	2.07	1.9E-03	0.0162
SERPINB3	2.06	8.0E-03	0.0381
HAS3	2.06	3.2E-03	0.0220
UGCG	2.05	5.1E-03	0.0289
XKRX	2.05	8.5E-03	0.0393
GALNT18	2.04	2.8E-04	0.0062
LGALS7B	2.03	7.3E-03	0.0361
SERPINF1	2.03	8.4E-03	0.0389
MAP1LC3A	2.03	2.5E-03	0.0189
FABP5	2.03	3.6E-04	0.0181
RDH16	2.02	9.1E-04	0.0111
SESN3	2.02	8.9E-03	0.0406
ACP5	2.02	1.2E-03	0.0126
INPP4B	2.02	4.7E-03	0.0275
KRT23	2.01	4.7E-03	0.0273
UBASH3B	2.01	6.0E-03	0.0321
PON2	2.01	9.5E-05	0.0039
PIK3R2	-2.00	1.0E-04	0.0040
GCLC	-2.00	1.0E-03	0.0120
TTLL12	-2.00	3.6E-03	0.0235
MT1E	-2.00	3.7E-03	0.0240
ARHGEF16	-2.01	1.4E-04	0.0045
SUOX	-2.01	2.9E-04	0.0064
COBLL1	-2.01	3.7E-04	0.0070
MATN2	-2.01	3.5E-04	0.0069
C6ORF136	-2.01	4.4E-05	0.0029
SMO	-2.01	8.8E-05	0.0038
SERTAD4	-2.01	5.6E-04	0.0087
RAVER2	-2.02	2.5E-05	0.0024
DEPTOR	-2.02	3.1E-04	0.0066
MECOM	-2.02	2.1E-03	0.0173
MRPL23	-2.02	6.9E-04	0.0097
DCAKD	-2.03	2.3E-04	0.0057
FAM3B	-2.03	6.8E-03	0.0347
TMEM109	-2.04	2.5E-04	0.0059
P2RY1	-2.04	6.8E-03	0.0153
CARM1	-2.04	7.4E-04	0.0101

ABLIM2	-2.05	2.0E-03	0.0168
OAT	-2.05	4.3E-04	0.0139
PGAP3	-2.05	1.6E-08	0.0001
CDK10	-2.06	1.4E-03	0.0139
CIDEB	-2.06	4.1E-05	0.0028
CX3CL1	-2.06	4.3E-03	0.0259
C17ORF97	-2.07	3.1E-03	0.0215
BOC	-2.07	8.5E-05	0.0037
RAPGEFL1	-2.07	8.3E-05	0.0037
SLC7A5	-2.07	8.8E-04	0.0109
CMTM4	-2.07	3.6E-04	0.0042
DHRS3	-2.08	7.5E-05	0.0035
DGCR6L	-2.08	2.8E-04	0.0062
DGCR6	-2.08	3.8E-03	0.0293
PHKA2	-2.08	1.8E-04	0.0050
D2HGDH	-2.08	1.5E-03	0.0145
EPHX2	-2.09	1.2E-04	0.0042
MT1A	-2.09	5.0E-04	0.0082
KCTD3	-2.10	1.9E-03	0.0248
EPCAM	-2.11	2.2E-04	0.0243
NOTCH3	-2.11	2.4E-03	0.0184
SLC6A9	-2.11	3.0E-03	0.0214
SPSB1	-2.12	3.5E-05	0.0027
SLC1A4	-2.13	4.8E-06	0.0012
NFIC	-2.14	1.1E-03	0.0123
FLOT2	-2.14	2.1E-03	0.0169
SLC6A10P	-2.15	1.5E-04	0.0046
PTPRF	-2.15	4.4E-03	0.0263
NFIA	-2.15	1.2E-04	0.0042
CUTA	-2.15	1.5E-04	0.0046
ZNF462	-2.15	1.9E-05	0.0021
BLVRB	-2.15	1.2E-02	0.0489
DDAH1	-2.16	1.2E-04	0.0042
MYLIP	-2.17	4.7E-05	0.0029
SREBF1	-2.17	1.0E-04	0.0040
TNNI2	-2.18	1.2E-04	0.0042
EML3	-2.19	1.2E-04	0.0042
SMARCA2	-2.19	4.0E-05	0.0028
PLEKHH3	-2.20	4.9E-05	0.0029
DIS3L	-2.20	1.3E-04	0.0044
CRIP2	-2.20	3.5E-04	0.0069
COMTD1	-2.20	8.7E-04	0.0108
FAT2	-2.20	4.0E-03	0.0248
HIST1H4C	-2.20	6.6E-03	0.0338
EPN2	-2.20	4.8E-04	0.0080
TRIOBP	-2.21	3.9E-05	0.0027
KIAA1211L	-2.21	9.2E-04	0.0112
ALAD	-2.22	1.2E-03	0.0130
CBR1	-2.22	9.9E-04	0.0117
AKR1C3	-2.22	2.5E-03	0.0189
DENND4C	-2.22	4.8E-05	0.0029
BARD1	-2.22	1.6E-05	0.0019
NOL12	-2.23	1.6E-03	0.0151
ZSCAN18	-2.24	6.1E-05	0.0032
NUSAP1	-2.24	4.7E-04	0.0079
ATP6V0A4	-2.25	7.9E-03	0.0376

RALGPS1	-2.26	1.4E-04	0.0044
DTX2	-2.27	6.0E-04	0.0090
BTBD2	-2.28	2.2E-04	0.0055
PLLP	-2.29	1.2E-04	0.0019
PEMT	-2.29	5.9E-05	0.0032
FAR1	-2.29	1.4E-03	0.0138
HES1	-2.30	3.8E-05	0.0027
OLFML2A	-2.31	8.0E-04	0.0104
ECHDC2	-2.31	3.6E-04	0.0070
SLC9A3R1	-2.33	4.3E-03	0.0261
LINC01315	-2.34	4.3E-07	0.0003
TSPAN7	-2.35	2.3E-03	0.0069
SF3B3	-2.35	3.0E-04	0.0064
NBL1	-2.36	1.4E-03	0.0140
ZBTB42	-2.37	7.1E-05	0.0034
CHCHD10	-2.38	2.4E-04	0.0058
GSTT1	-2.38	7.9E-04	0.0104
MIR205	-2.38	2.1E-03	0.0172
AKR1C4	-2.39	7.3E-04	0.0099
ECHDC3	-2.39	2.9E-03	0.0207
EEF2K	-2.39	5.6E-05	0.0031
ITPRIPL2	-2.40	9.2E-05	0.0039
SLF2	-2.40	3.8E-06	0.0011
UGDH	-2.41	3.9E-04	0.0072
NUCKS1	-2.41	9.7E-05	0.0007
SFRP1	-2.43	8.3E-05	0.0037
HRASLS	-2.43	6.1E-05	0.0032
MAP3K1	-2.43	1.2E-04	0.0042
FRAS1	-2.45	8.2E-07	0.0005
DDR1	-2.46	3.2E-04	0.0066
PPARGC1A	-2.47	1.3E-04	0.0044
HMGB2	-2.47	3.8E-04	0.0071
AIM1L	-2.48	4.4E-04	0.0186
MGST2	-2.48	5.9E-04	0.0089
VAV3	-2.49	7.1E-05	0.0034
PTN	-2.49	1.1E-09	0.0000
BCKDHA	-2.51	3.4E-06	0.0011
CAPN5	-2.52	2.4E-04	0.0058
SAMD5	-2.52	5.7E-04	0.0087
CCND1	-2.52	2.0E-04	0.0052
DCXR	-2.53	1.8E-05	0.0020
CBR3	-2.54	5.3E-04	0.0084
NNAT	-2.56	6.6E-05	0.0033
SHMT1	-2.58	3.9E-03	0.0246
ZDHHC11	-2.60	4.2E-03	0.0259
SYT15	-2.62	4.7E-03	0.0275
WLS	-2.64	1.9E-04	0.0110
THEM6	-2.69	1.6E-05	0.0019
TKT	-2.71	1.1E-03	0.0122
FAM189A2	-2.74	3.4E-04	0.0068
ZNF395	-2.74	4.6E-05	0.0029
ELF3	-2.76	2.2E-03	0.0178
CEACAM1	-2.79	2.6E-03	0.0120
STOX2	-2.82	1.4E-04	0.0044
IGFBP3	-2.83	6.5E-03	0.0258
CYP11A1	-2.84	1.4E-06	0.0006

KRT31	-2.88	3.4E-03	0.0229
AMER1	-2.90	2.5E-06	0.0009
CUEDC1	-2.93	9.9E-04	0.0117
PAFAH1B3	-2.96	3.3E-03	0.0224
PAQR8	-2.98	1.2E-04	0.0042
RGMA	-3.04	1.3E-05	0.0019
SCIN	-3.13	7.5E-05	0.0035
MIR99AHG	-3.13	1.2E-05	0.0019
PAMR1	-3.14	4.4E-03	0.0264
OPLAH	-3.15	8.5E-08	0.0001
NMU	-3.16	1.1E-02	0.0455
NFIX	-3.20	1.3E-05	0.0019
CLDN23	-3.21	6.5E-06	0.0014
PTGR1	-3.25	8.7E-04	0.0339
AKR1A1	-3.26	2.7E-05	0.0014
ROR1	-3.29	6.7E-05	0.0033
HMGCS1	-3.34	1.8E-05	0.0020
MAOB	-3.43	1.1E-05	0.0018
CYP4X1	-3.43	7.4E-06	0.0134
SLC25A23	-3.49	2.9E-04	0.0064
ZNF296	-3.50	1.2E-04	0.0042
FAM117B	-3.53	4.5E-07	0.0003
TGFBR3	-3.53	9.6E-05	0.0039
ZBTB7C	-3.54	3.7E-06	0.0011
NFIB	-3.63	3.3E-06	0.0011
CYP4F12	-3.65	1.9E-03	0.0162
BCL11A	-3.65	4.0E-05	0.0028
FGFR3	-3.69	1.0E-04	0.0040
ANKRD20A1	-3.74	9.3E-07	0.0005
DAPL1	-3.76	8.7E-03	0.0399
AKR1C2	-3.85	3.7E-05	0.0027
KRT8	-3.87	1.2E-05	0.0019
MAOA	-3.93	2.5E-04	0.0059
PITX2	-3.97	1.2E-03	0.0035
COX7A1	-3.98	2.9E-05	0.0025
ASS1	-4.00	1.6E-03	0.0077
GPD1L	-4.01	6.4E-07	0.0004
PGD	-4.15	1.6E-06	0.0007
TF	-4.32	1.2E-04	0.0042
MT1X	-4.32	1.8E-04	0.0050
WNK4	-4.39	8.0E-04	0.0104
E2F2	-4.48	4.0E-05	0.0028
CLDN7	-4.73	2.1E-03	0.0173
ALDH3A1	-4.78	1.0E-02	0.0436
ALDH3A2	-4.84	2.7E-07	0.0192
PHGDH	-4.86	1.1E-05	0.0018
ETNK2	-5.13	4.8E-05	0.0029
RBM20	-5.18	2.3E-04	0.0058
CYP3A5	-5.56	4.9E-05	0.0029
MAMDC2	-6.56	1.1E-03	0.0122
KRT3	-6.70	5.3E-03	0.0294
MT1G	-8.75	2.9E-06	0.0010
CES1	-9.31	1.6E-03	0.0147
KRT4	-11.06	7.0E-04	0.0097
MUC21	-12.29	1.7E-02	0.0318
