# Supplementary material for: Gene signatures associated with barrier dysfunction and infection in oral lichen planus identified by analysis of transcriptomic data
Source: PLoS One. 2021 Sep 10;16(9):e0257356. doi: 10.1371/journal.pone.0257356 (PMC8432868; doi:10.1371/journal.pone.0257356)
Supplement: S3 Table — (PDF) [file pone.0257356.s003.pdf]

**S3 Table. Differentially expressed genes (DEGs) in the mucosa whole dataset**

| Gene symbol | Fold-change | *p*-value | *q*-value |
|---|---|---|---|
| CDSN | 6.57 | 3.4E-02 | 0.43 |
| CXCL13 | 6.54 | 1.6E-02 | 0.41 |
| WFDC12 | 6.13 | 4.2E-02 | 0.44 |
| SLC6A14 | 5.90 | 4.7E-02 | 0.44 |
| CD177 | 3.67 | 3.0E-02 | 0.48 |
| POSTN | 3.43 | 2.4E-02 | 0.42 |
| ADAMDEC1 | 3.26 | 3.3E-02 | 0.43 |
| SLAMF7 | 2.99 | 4.8E-03 | 0.40 |
| ASPN | 2.78 | 4.8E-02 | 0.44 |
| KLK12 | 2.62 | 2.8E-02 | 0.43 |
| SPRR2D | 2.53 | 4.4E-02 | 0.44 |
| MIR142 | 2.52 | 3.6E-02 | 0.43 |
| LAMP3 | 2.46 | 4.3E-02 | 0.44 |
| KRT24 | 2.30 | 4.0E-02 | 0.43 |
| AIM2 | 2.19 | 9.8E-03 | 0.40 |
| DPP4 | 2.18 | 2.7E-02 | 0.43 |
| PARP15 | 2.16 | 2.9E-02 | 0.43 |
| NELL2 | 2.13 | 4.0E-02 | 0.43 |
| LINC01559 | 2.08 | 4.1E-02 | 0.44 |
| RHOF | 2.06 | 6.1E-03 | 0.40 |
| VCAM1 | 2.06 | 4.9E-02 | 0.44 |
| LAYN | 2.01 | 6.4E-04 | 0.40 |
| SLC27A6 | -2.03 | 4.9E-02 | 0.44 |
| SNRPN | -2.11 | 2.9E-02 | 0.43 |
| AKR1C2 | -2.12 | 1.4E-02 | 0.40 |
| MUC4 | -2.20 | 2.2E-02 | 0.42 |
| UGT1A3/UGT1A1/UGT1A4/UGT1A9/UGT1A5/UGT1A6/UGT1A7/UGT1A8/UGT1A10 | -2.47 | 2.4E-02 | 0.42 |
| GSTA1 | -2.78 | 2.7E-02 | 0.43 |
| FAM3B | -3.34 | 3.8E-02 | 0.43 |
| GABRP | -3.50 | 1.2E-02 | 0.40 |
| DDX3Y | -4.02 | 3.3E-02 | 0.43 |
| RPS4Y1 | -4.23 | 2.9E-02 | 0.43 |
| EIF1AY | -5.36 | 2.4E-02 | 0.42 |
