# Supplementary material for: Gene signatures associated with barrier dysfunction and infection in oral lichen planus identified by analysis of transcriptomic data
Source: PLoS One. 2021 Sep 10;16(9):e0257356. doi: 10.1371/journal.pone.0257356 (PMC8432868; doi:10.1371/journal.pone.0257356)
Supplement: S4 Table — (PDF) [file pone.0257356.s004.pdf]

S4 Table. Differentially expressed genes (DEGs) in the mucosa partial dataset

Gene symbol	Fold-change	*p*-value	*q*-value
SLC6A14	35.48	8.3E-04	0.0416
CCL18	30.49	1.6E-04	0.0270
CDSN	28.85	8.4E-04	0.0416
LCE3E	25.54	1.5E-04	0.0266
CXCL13	21.56	6.9E-05	0.0217
IL36G	17.00	5.1E-05	0.0201
KRT75	15.16	1.5E-05	0.0183
IL7R	11.93	7.0E-05	0.0219
TMEM45A	11.37	8.1E-05	0.0239
KRT17	10.57	6.7E-04	0.0388
ABCA12	10.55	3.2E-05	0.0183
S100A7A	9.98	1.3E-03	0.0482
MMP9	9.31	8.8E-04	0.0416
IGKC	9.30	7.2E-04	0.0397
SPINK6	8.31	2.9E-04	0.0323
GSDMA	7.89	1.4E-06	0.0078
SELL	7.73	1.3E-03	0.0481
F2RL2	7.60	2.4E-05	0.0183
LAMC2	7.58	1.7E-04	0.0270
PLA2G7	7.27	9.3E-04	0.0420
TNC	7.21	1.1E-03	0.0457
MS4A1	6.76	8.9E-04	0.0417
SLAMF6	6.48	1.2E-03	0.0459
IL2RG	6.19	1.8E-04	0.0271
IL2RA	6.00	4.3E-04	0.0341
PIM2	5.94	9.8E-04	0.0427
FPR3	5.89	8.8E-04	0.0416
FCGR2A	5.89	5.8E-04	0.0368
TMEM176A	5.85	6.1E-05	0.0208
CD27	5.78	6.2E-04	0.0380
MIR142	5.73	2.3E-04	0.0300
BIRC3	5.72	5.6E-05	0.0207
CCL19	5.69	5.8E-05	0.0208
SEL1L3	5.69	2.0E-04	0.0284
SLAMF7	5.67	5.7E-04	0.0368
IFI30	5.56	5.8E-04	0.0368
LAMP3	5.55	1.4E-04	0.0257
LYZ	5.53	4.8E-04	0.0347
C2	5.47	1.9E-05	0.0183
PTPRC	5.42	8.0E-04	0.0413
LCP2	5.28	4.6E-05	0.0195
CD80	5.28	3.3E-04	0.0337
CCR1	5.25	1.5E-03	0.0497
SLAMF8	5.24	1.6E-05	0.0183
TNFRSF9	5.19	1.3E-03	0.0485
GBP5	5.17	6.5E-04	0.0387
CSF2RB	5.17	4.2E-04	0.0341
CCL13	5.16	4.1E-04	0.0341
AOAH	5.06	2.2E-04	0.0294
GLIPR1	5.03	9.1E-04	0.0420
CD28	5.02	2.8E-04	0.0323
IRF8	4.98	8.1E-04	0.0413
C3AR1	4.98	8.5E-04	0.0416

NRP2	4.96	6.7E-04	0.0390
MS4A4A	4.91	4.3E-04	0.0341
TSPAN11	4.90	5.3E-04	0.0360
CYBB	4.81	1.1E-03	0.0453
IKZF3	4.79	1.0E-03	0.0433
UBD	4.76	3.9E-04	0.0341
FCGR1CP/FCGR1B/FCGR1A	4.76	7.6E-04	0.0412
CD53	4.72	6.8E-04	0.0390
PAPSS2	4.69	4.7E-04	0.0347
BICC1	4.67	4.6E-04	0.0345
PDCD1LG2	4.67	2.8E-04	0.0323
CD84	4.66	6.6E-04	0.0387
MPEG1	4.65	7.5E-04	0.0409
MRC1	4.57	7.2E-04	0.0397
SERPINB9	4.47	8.0E-04	0.0413
TENM2	4.47	3.2E-04	0.0335
CCL22	4.41	8.0E-04	0.0413
C1QC	4.41	1.2E-03	0.0473
RAC2	4.36	9.3E-05	0.0246
SLC39A8	4.34	1.4E-03	0.0494
MS4A14/MS4A7	4.33	2.5E-05	0.0183
KCNA3	4.32	9.8E-04	0.0427
DDX60L	4.32	1.3E-03	0.0485
NCKAP1L	4.31	1.6E-04	0.0270
PLEK	4.29	6.9E-04	0.0391
LIPN	4.28	3.6E-04	0.0340
ANPEP	4.23	4.5E-04	0.0345
PTPN22	4.21	1.4E-04	0.0263
CCL21	4.19	5.5E-04	0.0363
ADAM19	4.17	5.7E-04	0.0368
GPR65	4.12	3.5E-04	0.0340
FMOD	4.10	6.4E-04	0.0384
SELPLG	4.08	8.2E-04	0.0413
CD4	4.07	7.4E-04	0.0404
GMFG	4.04	1.6E-04	0.0270
F2R	4.04	1.4E-04	0.0257
CD209/CLEC4M	4.03	4.6E-04	0.0345
TRAF3IP3	4.00	4.0E-04	0.0341
PARP15	3.99	4.4E-05	0.0193
CD163	3.98	2.4E-05	0.0183
CTLA4	3.97	3.6E-04	0.0340
CECR1	3.97	1.0E-03	0.0445
DOCK2	3.94	3.3E-04	0.0337
LCP1	3.90	6.4E-04	0.0384
LPXN	3.88	1.4E-05	0.0183
GLRX	3.84	2.9E-05	0.0183
CD86	3.84	7.7E-04	0.0412
ADAP2	3.80	1.3E-04	0.0252
LAPTM5	3.71	1.2E-03	0.0473
CXORF21	3.68	2.0E-04	0.0284
SLC41A2	3.66	9.3E-04	0.0420
STAT4	3.65	7.4E-04	0.0404
PLTP	3.60	4.2E-04	0.0341
TGFBI	3.59	2.5E-04	0.0309
LTBP1	3.57	7.3E-04	0.0403
LYN	3.57	1.2E-03	0.0473

CYTH4	3.53	1.2E-04	0.0252
RASSF2	3.52	4.9E-04	0.0347
CSF2RA	3.49	2.6E-04	0.0314
GPR132	3.48	3.4E-04	0.0337
TNFRSF1B	3.47	3.7E-04	0.0341
ETS1	3.44	6.5E-04	0.0387
CD37	3.43	8.1E-04	0.0413
MICAL2	3.43	9.2E-04	0.0420
BIN2	3.41	3.4E-04	0.0337
THEMIS2	3.41	4.8E-04	0.0347
ITM2C	3.39	6.9E-04	0.0391
DAB2	3.38	3.3E-04	0.0337
HLA-DMB	3.35	8.3E-04	0.0416
CCND2	3.35	4.2E-04	0.0341
TLR8	3.34	4.3E-04	0.0341
HLA-DOB	3.31	1.0E-04	0.0252
GALNT6	3.30	3.6E-06	0.0100
C1QA	3.29	9.4E-04	0.0420
JAK3	3.29	5.8E-05	0.0208
TTYH3	3.28	2.7E-04	0.0320
CTSS	3.27	1.4E-03	0.0487
P2RY8	3.27	1.5E-03	0.0500
ANTXR2	3.23	1.2E-03	0.0473
RUNX2	3.22	5.3E-04	0.0360
ITGA4	3.21	2.3E-04	0.0300
APBB1IP	3.21	2.2E-04	0.0293
KLK9	3.21	3.5E-05	0.0183
ORAI2	3.20	1.7E-04	0.0270
CST7	3.19	1.4E-03	0.0494
F5	3.17	8.5E-04	0.0416
PTPRJ	3.17	8.5E-04	0.0416
TFEC	3.16	1.3E-04	0.0252
C1ORF162	3.11	8.6E-04	0.0416
MS4A6A	3.09	7.2E-04	0.0397
FCGR2C/FCGR2B	3.08	4.2E-04	0.0341
ADTRP	3.08	3.5E-04	0.0340
ERP27	3.08	3.4E-05	0.0183
LAIR1	3.08	3.5E-04	0.0340
SP110	3.08	7.2E-04	0.0397
CTSH	3.07	1.4E-03	0.0494
TMEM176B	3.07	3.6E-04	0.0340
RHOF	3.06	9.7E-04	0.0427
PAG1	3.03	4.2E-04	0.0341
LOC105369230/HLA-DRB6/HLA-DRB5/HLA-DRB4/HLA-DRB3/HLA-DRB1/HLA-DQB1	3.03	1.2E-04	0.0252
PYHIN1	3.02	9.1E-04	0.0420
MYO1G	3.02	2.4E-04	0.0303
RASSF4	2.99	9.2E-05	0.0246
SLA	2.96	6.2E-04	0.0380
SH2D2A	2.95	1.1E-03	0.0457
WDFY4	2.95	3.0E-04	0.0324
TMEM106A	2.95	6.6E-05	0.0251
ARPC1B	2.95	1.1E-03	0.0449
CIB2	2.94	4.2E-04	0.0341
FAM65B	2.94	4.2E-04	0.0341
FCGR2B	2.94	1.4E-03	0.0494

STK17A	2.93	8.6E-04	0.0416
HCLS1	2.91	1.9E-04	0.0282
KLHL6	2.89	6.2E-04	0.0381
SYT11	2.88	4.1E-04	0.0341
HLA-DRA/HLA-DQA1	2.86	1.1E-03	0.0417
PRKCB	2.86	9.2E-04	0.0420
PREX1	2.86	1.3E-04	0.0252
SLC15A3	2.85	5.8E-04	0.0368
HAPLN3	2.83	2.5E-05	0.0183
GPRIN3	2.80	9.1E-04	0.0420
PGLYRP4	2.80	6.2E-04	0.0380
SDK1	2.80	1.4E-03	0.0486
SPOCK2	2.78	1.1E-03	0.0449
COTL1	2.74	7.6E-05	0.0233
RASA3	2.74	6.5E-04	0.0387
ARRDC4	2.73	3.6E-04	0.0340
RNASE6	2.70	9.3E-04	0.0420
C1R	2.69	4.3E-04	0.0341
CTSZ	2.69	1.6E-04	0.0267
TAP1	2.69	8.8E-04	0.0416
CPVL	2.69	9.8E-04	0.0427
TRAF1	2.68	1.3E-03	0.0486
C12ORF75	2.67	3.5E-05	0.0183
GPR137B	2.67	1.7E-04	0.0270
FERMT3	2.65	4.3E-04	0.0341
SLC39A6	2.64	2.9E-05	0.0183
PARP12	2.63	5.8E-04	0.0368
SERPING1	2.62	2.4E-04	0.0302
NEK6	2.62	1.5E-04	0.0266
SMAP2	2.61	7.8E-04	0.0413
SASH3	2.60	2.9E-04	0.0323
CMTM7	2.60	2.8E-04	0.0323
GRK3	2.59	1.3E-03	0.0478
RFTN1	2.59	1.2E-04	0.0252
CD74/ROS1	2.57	9.2E-05	0.0246
RAB29	2.57	9.6E-05	0.0251
INPP5D	2.56	5.2E-04	0.0356
ANKRD44	2.56	1.3E-03	0.0485
TGFB3	2.56	8.4E-04	0.0416
GIMAP1-GIMAP5/GIMAP5	2.55	1.3E-03	0.0486
HAVCR2	2.55	3.8E-04	0.0341
KIAA0040	2.54	1.1E-04	0.0252
FAM69A	2.52	5.4E-04	0.0363
CLSTN3	2.52	3.8E-04	0.0341
STAB1	2.52	3.0E-04	0.0324
P2RX4	2.51	3.8E-04	0.0341
CSGALNACT2	2.51	8.0E-04	0.0413
BTK	2.50	8.0E-04	0.0413
EMP3	2.50	9.9E-04	0.0427
ADGRE5	2.50	3.9E-04	0.0341
SFXN3	2.49	1.1E-03	0.0449
HLA-F/HLA-B	2.49	3.7E-04	0.0296
PLSCR1	2.49	2.8E-04	0.0323
CCDC88A	2.48	1.3E-04	0.0252
CDYL2	2.48	1.0E-04	0.0251
FMNL1	2.47	6.1E-05	0.0208

ADAM28	2.46	7.6E-04	0.0410
PLOD3	2.46	6.6E-04	0.0387
NABP1	2.45	1.1E-03	0.0449
ZYX	2.44	1.4E-03	0.0491
CD163L1	2.44	1.3E-03	0.0486
DOC2B	2.43	3.1E-05	0.0324
NAV1	2.43	1.1E-04	0.0252
STK10	2.41	8.8E-06	0.0168
GNG2	2.40	7.0E-04	0.0395
ARHGAP25	2.40	4.6E-04	0.0345
VAV1	2.39	1.0E-03	0.0433
APOL1	2.38	4.5E-04	0.0345
IFITM1	2.36	1.1E-03	0.0457
UBASH3A	2.35	3.8E-04	0.0341
CDC42EP3	2.34	1.5E-03	0.0497
ATP8B2	2.34	1.1E-03	0.0457
SP140	2.34	1.4E-04	0.0257
FAR2	2.33	1.2E-03	0.0473
TLR4	2.33	3.5E-04	0.0340
IL18R1	2.31	9.4E-04	0.0420
MAN2B1	2.31	2.0E-05	0.0183
C11ORF24	2.30	4.5E-04	0.0345
FMNL3	2.29	2.9E-04	0.0324
LITAF	2.29	2.7E-04	0.0323
MARCH1	2.28	8.1E-04	0.0413
TMEM173	2.28	1.4E-03	0.0492
ST3GAL5	2.27	6.3E-04	0.0384
TMEM184B	2.25	1.4E-03	0.0490
FCHSD2	2.25	7.8E-04	0.0413
ACOT9	2.23	5.3E-04	0.0360
LOC107987457/PPP1R18	2.23	2.2E-04	0.0294
FAM167A	2.23	8.8E-04	0.0416
DLGAP1-AS1	2.23	3.8E-05	0.0183
TCN2	2.23	2.0E-04	0.0285
C1RL	2.23	2.3E-04	0.0300
STAT2	2.22	1.9E-04	0.0278
PSTPIP2	2.21	1.3E-04	0.0252
GNS	2.21	1.0E-03	0.0430
KCND3	2.21	9.0E-04	0.0418
ZNFX1	2.21	9.9E-05	0.0251
FEZ1	2.20	1.2E-03	0.0464
LAYN	2.19	1.8E-05	0.0183
RASAL3	2.18	4.0E-04	0.0341
STX2	2.18	8.0E-04	0.0413
ARHGEF6	2.17	1.3E-03	0.0482
TMEM256-PLSCR3/PLSCR3	2.17	1.1E-03	0.0456
FXYD5	2.15	4.9E-04	0.0348
STS	2.15	2.3E-04	0.0300
DFNA5	2.15	7.7E-04	0.0412
ENAH	2.14	2.2E-06	0.0083
GLB1	2.11	3.7E-05	0.0183
DPYSL3	2.11	7.1E-04	0.0395
CMTM3	2.10	2.0E-04	0.0282
PITPNC1	2.10	3.6E-04	0.0340
WAS	2.10	3.2E-04	0.0332
CELF2	2.10	1.5E-04	0.0266

NFATC2	2.10	9.6E-04	0.0426
RNF130	2.10	5.7E-04	0.0368
RNF213	2.09	1.1E-03	0.0457
MIR15A	2.08	1.4E-03	0.0487
GAB3	2.07	3.0E-04	0.0324
SFMBT2	2.07	1.1E-03	0.0449
SDC3	2.06	1.9E-04	0.0278
INPP4A	2.06	1.8E-04	0.0275
TRPV3	2.06	3.0E-04	0.0324
INPP4B	2.05	3.1E-04	0.0330
PCDHB16	2.05	5.6E-04	0.0368
PLBD2	2.04	4.8E-04	0.0347
TAPBP	2.01	4.9E-04	0.0368
UBASH3B	2.01	1.1E-04	0.0252
DNAJC10	2.01	8.6E-04	0.0416
ARHGAP18	2.01	6.1E-04	0.0375
FMNL2	2.01	1.1E-03	0.0457
TMEM263	2.01	1.2E-03	0.0463
TINCR	-2.01	6.5E-04	0.0387
IL1RL2	-2.02	9.9E-04	0.0427
PARD3	-2.03	1.1E-03	0.0449
LOC728554/THOC3	-2.03	2.3E-03	0.0491
TICRR	-2.06	6.0E-04	0.0374
MYH14	-2.08	1.5E-03	0.0497
LOC727751/GOLGA2P3Y/GOLGA2P7/GOLGA2P2Y/GOLGA2P10	-2.10	4.4E-05	0.0239
PRDX5	-2.11	1.1E-03	0.0457
BHLHE40	-2.11	1.4E-03	0.0494
PLD1	-2.14	8.1E-04	0.0413
WNK4	-2.15	8.4E-04	0.0416
AIM1L	-2.19	3.3E-04	0.0337
PTPRF	-2.20	2.7E-04	0.0320
KLF8	-2.22	1.5E-04	0.0266
PIR-FIGF/PIR	-2.24	5.1E-04	0.0356
TTTY14	-2.25	3.1E-04	0.0330
SNORA59A/SNORA59B	-2.30	1.1E-03	0.0449
C15ORF59	-2.31	5.9E-04	0.0368
PLEKHG6	-2.32	3.7E-04	0.0340
P2RY2	-2.40	9.5E-04	0.0421
BCL11A	-2.41	4.6E-04	0.0345
SLC24A3	-2.46	4.0E-04	0.0341
PLEKHA7	-2.49	3.8E-05	0.0183
NLGN4Y	-2.55	4.7E-04	0.0345
IKZF2	-2.55	1.4E-03	0.0492
MGST2	-2.56	4.0E-04	0.0341
FRAS1	-2.60	3.5E-04	0.0340
CYP11A1	-2.72	5.4E-04	0.0363
CBR1	-2.74	2.6E-04	0.0314
PLLP	-2.77	5.3E-06	0.0131
PDCD4	-2.78	4.1E-04	0.0341
PGD	-2.92	6.9E-04	0.0391
FGFR3	-3.00	1.4E-03	0.0494
LOC654780	-3.02	2.8E-05	0.0183
PAIP2B/VPS36	-3.09	1.1E-03	0.0450
MAOA	-3.12	1.1E-05	0.0168
CYP4F12	-3.21	6.9E-04	0.0391

ZBTB7C	-3.34	1.4E-03	0.0492
ST6GAL2	-3.47	3.6E-05	0.0183
PTN	-3.95	5.5E-05	0.0207
HMGCS1	-4.21	2.1E-04	0.0291
RAPGEFL1	-4.43	1.3E-03	0.0478
HLF	-4.57	4.4E-04	0.0343
KDM5D	-4.78	9.8E-06	0.0168
SLC4A4	-4.83	8.4E-04	0.0416
ALDH3A1	-5.52	1.1E-03	0.0457
KRT15	-7.47	9.4E-04	0.0421
SCIN	-8.57	6.5E-05	0.0215
USP9Y	-8.74	2.8E-06	0.0090
UTY	-10.05	2.9E-05	0.0183
ETNK2	-12.06	1.5E-04	0.0266
DDX3Y	-12.17	6.6E-04	0.0387
RPS4Y1	-12.46	6.2E-08	0.0014
EIF1AY	-17.98	1.4E-03	0.0491
