# Supplementary material for: Gene signatures associated with barrier dysfunction and infection in oral lichen planus identified by analysis of transcriptomic data
Source: PLoS One. 2021 Sep 10;16(9):e0257356. doi: 10.1371/journal.pone.0257356 (PMC8432868; doi:10.1371/journal.pone.0257356)
Supplement: S5 Table — (PDF) [file pone.0257356.s005.pdf]

Upregulated, Downregulated

S5 Table. Gene Ontology biological process terms enriched in the epithelium whole dataset

Term	Count	p-value	Gene
epidermis development	13	6.7E-11	S100A7, ALDH3A2, CALML5, KLK5, KLK7, KRT16, KRT17, KRT31, LCE3D, SPRR1B, SPRR2B, SPRR2E, SPRR2G
keratinocyte differentiation	12	3.2E-10	S100A7, FLG, KRT10, KRT16, LCE3A, LCE3D, LCE3E, LOR, SPRR1B, SPRR2B, SPRR2E, SPRR2G
keratinization	10	1.3E-09	KRT16, KRT17, LCE3A, LCE3D, LCE3E, LOR, SPRR1B, SPRR2B, SPRR2E, SPRR2G
peptide cross-linking	9	4.0E-08	COL3A1, LCE3A, LCE3D, LCE3E, LOR, SPRR1B, SPRR2B, SPRR2E, SPRR2G
collagen catabolic process	6	5.1E-04	CTSL, COL1A2, COL3A1, COL4A1, COL5A2, COL6A3
cellular response to platelet-derived growth factor stimulus	4	7.8E-04	ERRFI1, HYAL1, PDGFRB, PTN
establishment of skin barrier	4	7.8E-04	ALOX12B, ALOXE3, FLG, KRT16
extracellular matrix organization	9	9.8E-04	C6ORF15, COL1A2, COL3A1, COL4A1, COL5A2, COL6A3, CRISPLD2, LUM, TNC
oxidation-reduction process	16	1.2E-03	HIGD1A, ALDH3A2, AKR1A1, AKR1B15, ALOX12B, ALOXE3, CYP11A1, CYP4F12, CYP4F22, GPD1L, MAOA, MAOB, PGD, PHGDH, QSOX1, RDH12
cellular response to amino acid stimulus	5	1.4E-03	ASS1, COL1A2, COL3A1, COL4A1, COL5A2
hyaluronan biosynthetic process	3	2.1E-03	CEMIP, HYAL1, IL1B
skin development	4	5.1E-03	FRAS1, ASPRV1, COL3A1, COL5A2
collagen fibril organization	4	7.5E-03	COL1A2, COL3A1, COL5A2, LUM
negative regulation of endopeptidase activity	6	8.3E-03	WFDC5, COL6A3, FETUB, PI3, SERPINB7, SERPINE2
epithelium morphogenesis	3	8.9E-03	FRAS1, CA2, KRT16
defense response to Gram-positive bacterium	5	1.2E-02	CD36, C10ORF99, DEFB4A, HIST1H2BD, RNASE7
response to drug	9	1.4E-02	HMGCS1, PPARGC1A, ASS1, CCND1, DUSP6, GGH, LCN2, MAOB, PTN
response to estradiol	5	1.5E-02	ASS1, CCND1, IFI27, PDGFRB, PTN
positive regulation of biosynthetic process of antibacterial peptides active against Gram-positive bacteria	2	2.0E-02	DEFB103A, DEFB103B
arachidonic acid metabolic process	3	2.7E-02	ALOX12B, ALOXE3, CYP4F12
response to estrogen	4	2.9E-02	CA2, CCND1, PDGFRB, RCAN1
positive regulation of antibacterial peptide production	2	3.1E-02	KLK5, KLK7
response to mycotoxin	2	3.1E-02	ASS1, LCN2
negative regulation of membrane potential	2	3.1E-02	BNIP3, PTN
innate immune response	10	3.4E-02	S100A12, S100A7, DEFB103A, DEFB103B, DEFB4A, IL36RN, IL36G, KRT16, LCN2, RNASE7
heart development	6	4.1E-02	ADAP2, RBM20, COL3A1, FLRT3, GJA1, PTN
liver development	4	4.1E-02	HMGCS1, ASS1, PTN, TGFBR3
negative regulation of sodium ion transport	2	4.1E-02	WNK4, SERPINE2

brain development	6	4.7E-02	HMGCS1, COL4A1, EML1, PHGDH, PAFAH1B3, SLC23A1
