# Supplementary material for: Gene signatures associated with barrier dysfunction and infection in oral lichen planus identified by analysis of transcriptomic data
Source: PLoS One. 2021 Sep 10;16(9):e0257356. doi: 10.1371/journal.pone.0257356 (PMC8432868; doi:10.1371/journal.pone.0257356)
Supplement: S6 Table — (PDF) [file pone.0257356.s006.pdf]

Upregulated, Downregulated

S6 Table. Gene Ontology biological process terms enriched in the epithelium partial dataset

Term	Count	p-value	Gene
epidermis development	23	2.7E-17	ELF3, S100A7, UGCG, ALDH3A2, CALML5, CST6, FABP5, FLOT2, KLK5, KLK7, KRT16, KRT17, KRT31, LAMA3, LCE3D, SPRR1B, SPRR2A, SPRR2B, SPRR2D, SPRR2E, SPRR2F, SPRR2G, TGM5
keratinization	14	5.2E-11	CDH3, KRT16, KRT17, LCE3A, LCE3D, LCE3E, LOR, SPRR1B, SPRR2A, SPRR2B, SPRR2D, SPRR2E, SPRR2F, SPRR2G
keratinocyte differentiation	16	2.3E-10	S100A7, AKR1C3, FLG, KRT10, KRT16, LCE3A, LCE3D, LCE3E, LOR, SPRR1B, SPRR2A, SPRR2B, SPRR2D, SPRR2E, SPRR2F, SPRR2G
peptide cross-linking	13	1.4E-09	COL3A1, LCE3A, LCE3D, LCE3E, LOR, SPRR1B, SPRR2A, SPRR2B, SPRR2D, SPRR2E, SPRR2F, SPRR2G, TGM5
oxidation-reduction process	35	1.7E-06	HIGD1A, UGDH, ALDH3A1, ALDH3A2, AKR1A1, AKR1C2, AKR1C3, AKR1C4, ALOX12B, ALOXE3, BLVRB, BCKDHA, CBR1, CBR3, CYP11A1, CYP2C18, CYP4F12, CYP4F22, CYP4X1, DHRS3, DCXR, FAR1, GPD1L, MTHFD1L, MAOA, MAOB, PAM, PGD, PHGDH, PTGR1, QSOX1, RDH12, RDH16, SESN3, SMOX
extracellular matrix organization	17	1.7E-05	DNAJB6, ELF3, COMP, C6ORF15, COL1A2, COL3A1, COL4A1, COL6A3, CRISPLD2, DDR1, FBN2, HPSE, LAMA3, LUM, OLFML2A, TNC, TGFBI
cellular response to cadmium ion	6	3.5E-05	AKR1C3, HMOX1, MT1A, MT1E, MT1G, MT1X
response to drug	19	3.4E-04	HMGCS1, PPARGC1A, ALDH3A1, ALAD, ARG1, ASS1, CDH3, CCND1, DUSP6, HMGB2, LCN2, MAOB, PAM, PEMT, PTN, SFRP1, SREBF1, TGFA, VAV3
aging	13	5.5E-04	PPARGC1A, ALDH3A1, ARG1, ASS1, CARM1, COL3A1, GJB6, GCLC, KRT16, LITAF, P2RY1, SERPINF1, SREBF1
epithelial cell differentiation	8	1.3E-03	ELF3, AKR1C2, CBR1, CES1, COL4A1, DLX5, KRT3, KRT4
cellular response to cobalt ion	3	1.6E-03	BNIP3, TIGAR, SERPINF1
liver development	8	1.8E-03	HMGCS1, WNT4, ARG1, ASS1, HES1, PTN, PRKCSH, TGFBR3
response to vitamin E	4	1.9E-03	HMGCS1, ALAD, ARG1, CCND1
cellular response to insulin stimulus	8	2.2E-03	DENND4C, ERRFI1, RAB31, CEACAM1, EEF2K, GCLC, MYO5A, PIK3R2
cell adhesion	22	3.0E-03	BOC, CX3CL1, DGCR6, FAT1, THY1, CDH3, CEACAM1, COMP, COL6A3, CNTNAP2, DDR1, FEZ1, FLOT2, HES1, HAS3, LAMA3, LPXN, PTPRF, SLURP1, TNC, THBS2, TGFBI
positive regulation of mitotic nuclear division	5	3.0E-03	IL1A, IL1B, NUSAP1, PDGFRB, TGFA
cellular response to jasmonic acid stimulus	3	3.2E-03	AKR1C2, AKR1C3, AKR1C4

cellular response to amino acid stimulus	6	4.8E-03	CEBPB, ASS1, COL1A2, COL3A1, COL4A1, SESN3
cellular response to starvation	6	4.8E-03	WNT4, AKR1C3, CTSV, HSPA8, SFRP1, SREBF1
negative regulation of cell migration	8	7.2E-03	CX3CL1, SLC9A3R1, THY1, WNT4, KRT16, PTN, SLURP1, SFRP1
cellular response to prostaglandin D stimulus	3	7.8E-03	AKR1C2, AKR1C3, TNC
positive regulation of transforming growth factor beta1 production	3	7.8E-03	CX3CL1, LUM, SERPINB7
establishment of skin barrier	4	8.2E-03	ALOX12B, ALOXE3, FLG, KRT16
extracellular matrix disassembly	7	9.0E-03	HTRA1, CTSL, CTSV, FBN2, KLK7, LAMA3, PRSS2
negative regulation of growth	4	9.5E-03	MT1A, MT1E, MT1G, MT1X
cellular response to zinc ion	4	9.5E-03	MT1A, MT1E, MT1G, MT1X
response to zinc ion	5	9.9E-03	D2HGDH, ALAD, ARG1, ASS1, CA2
xenobiotic metabolic process	7	1.0E-02	S100A12, ALDH3A1, CES1, CYP2C18, CYP3A5, EPHX2, MGST2
negative regulation of cell adhesion	5	1.1E-02	CLDN7, LPXN, MUC21, TNC, TGFBI
hyaluronan biosynthetic process	3	1.1E-02	CEMIP, HAS3, IL1B
response to herbicide	3	1.1E-02	ALAD, ARG1, LCN2
cell-cell adhesion	14	1.2E-02	LIMA1, ARHGEF16, S100P, CEACAM1, COBLL1, EPCAM, EPN2, FSCN1, FLRT3, HSPA8, KIF5B, MACF1, MYO1B, NOTCH3
response to selenium ion	3	1.4E-02	ALAD, ARG1, MAOB
doxorubicin metabolic process	3	1.4E-02	AKR1C2, AKR1C3, AKR1C4
daunorubicin metabolic process	3	1.4E-02	AKR1C2, AKR1C3, AKR1C4
cellular response to vitamin D	3	1.4E-02	PTN, SFRP1, TNC
defense response to Gram-positive bacterium	7	1.5E-02	ACP5, C10ORF99, DEFB4A, HMGB2, HIST1H2BC, HIST1H2BD, RNASE7
digestion	6	1.6E-02	PPARGC1A, AKR1C2, NMU, PRSS2, PRSS3, SLC15A1
collagen catabolic process	6	1.7E-02	CTSL, COL1A2, COL3A1, COL4A1, COL6A3, PRSS2
cellular response to lipopolysaccharide	8	1.8E-02	CEBPB, PPARGC1A, PYCARD, ARG1, ASS1, HMGB2, LCN2, LITAF
arachidonic acid metabolic process	4	2.0E-02	JMJD7-PLA2G4B, ALOX12B, ALOXE3, CYP4F12
response to cadmium ion	4	2.0E-02	ALAD, ARG1, GCLC, PRNP
response to metal ion	3	2.2E-02	CUTA, MT1A, MT1X
negative regulation of endopeptidase activity	8	2.5E-02	WFDC5, COL6A3, FETUB, PI3, SERPINB3, SERPINB7, SERPINB8, SERPINF1
odontogenesis	4	2.5E-02	COL1A2, MYO5A, PITX2, PAM
cellular aldehyde metabolic process	3	2.7E-02	ALDH3A1, ALDH3A2, AKR1A1
cellular response to follicle-stimulating hormone stimulus	3	2.7E-02	HMGCS1, PPARGC1A, GCLC
negative regulation of DNA binding	4	2.8E-02	HMOX1, NFIB, SMO, ZNF462
positive regulation of release of cytochrome c from mitochondria	4	2.8E-02	BNIP3, PYCARD, CIDEB, MLLT11
positive regulation of vasodilation	4	3.0E-02	F2RL1, EPHX2, GJA1, HMOX1
cellular response to dexamethasone stimulus	4	3.0E-02	ERRFI1, ARG1, ASS1, SERPINF1
response to pH	3	3.1E-02	CA2, GJA1, PAM
retinol metabolic process	4	3.3E-02	AKR1C3, DHRS3, RBP1, RDH12
lung development	6	3.4E-02	ARG1, CRISPLD2, HES1, PTN, SLC23A1, SREBF1

regulation of protein localization	5	3.4E-02	AAK1, DNAJB6, FKBP1A, RAB9A, PRNP
protein localization to membrane	3	3.6E-02	RAB32, RAB38, CPE
response to arsenic-containing	3	3.6E-02	ALAD, GCLC, SERPINF1
prostaglandin metabolic process	3	3.6E-02	AKR1C2, AKR1C3, PTGR1
cytoskeleton organization	9	3.7E-02	THY1, KRT16, KRT31, KRT4, LOR, MAST4, MACF1, TUBB2A, TUBB3
osteoblast differentiation	7	3.7E-02	DLX5, GJA1, IGFBP3, SFRP1, SMO, TNC, TP53INP2
brown fat cell differentiation	4	3.9E-02	BNIP3, CEBPB, PPARGC1A, RGS2
wound healing	6	4.1E-02	CDH3, COL3A1, MACF1, PDGFRB, TNC, TGFA
odontogenesis of dentin-containing tooth	5	4.1E-02	CA2, NFIC, PITX2, SMO, TNC
defense response to Gram-negative bacterium	5	4.1E-02	PYCARD, S100A7, DEFB4A, HMGB2, RNASE7
cellular glucose homeostasis	3	4.2E-02	PPARGC1A, NUCKS1, PIK3R2
morphogenesis of an epithelium	3	4.2E-02	FRAS1, CA2, KRT16
thalamus development	3	4.2E-02	CNTNAP2, PTN, SMO
positive regulation of biosynthetic process of antibacterial peptides active against Gram-positive bacteria	2	4.7E-02	DEFB103A, DEFB103B
female gonad development	3	4.8E-02	WNT4, PITX2, SFRP1
response to copper ion	3	4.8E-02	IL1A, PAM, PRNP
phosphatidic acid biosynthetic process	4	4.9E-02	JMJD7-PLA2G4B, ABHD5, GPD1L, PLA2G4D
mitophagy	4	4.9E-02	BNIP3, GABARAPL2, PPARGC1A, MAP1LC3A
