# Supplementary material for: Gene signatures associated with barrier dysfunction and infection in oral lichen planus identified by analysis of transcriptomic data
Source: PLoS One. 2021 Sep 10;16(9):e0257356. doi: 10.1371/journal.pone.0257356 (PMC8432868; doi:10.1371/journal.pone.0257356)
Supplement: S7 Table — (PDF) [file pone.0257356.s007.pdf]

Upregulated, Downregulated

S7 Table. Gene Ontology biological process terms enriched in the mucosa whole dataset

Term	Count	p-value	Gene
chronic inflammatory response	2	1.5E-02	CXCL13, VCAM1
translational initiation	3	2.2E-02	DDX3Y, EIF1AY, RPS4Y1
response to hypoxia	3	3.3E-02	DPP4, POSTN, VCAM1
cell adhesion	4	4.0E-02	SLAMF7, CDSN, POSTN, VCAM1
