# Supplementary material for: Gene signatures associated with barrier dysfunction and infection in oral lichen planus identified by analysis of transcriptomic data
Source: PLoS One. 2021 Sep 10;16(9):e0257356. doi: 10.1371/journal.pone.0257356 (PMC8432868; doi:10.1371/journal.pone.0257356)
Supplement: S8 Table — (PDF) [file pone.0257356.s008.pdf]

Upregulated, Downregulated

S8 Table. Gene Ontology biological process terms enriched in the mucosa partial dataset

Term	Count	p-value	Gene
immune response	41	4.3E-18	CCL13, CCL18, CCL19, CCL21, CCL22, CCR1, CXCL13, CD27, CD4, CD74, CD86, ETS1, FCGR2B, GPR65, TAPBP, TNFRSF1B, TNFRSF9, WAS, ADGRE5, CTSS, C1QC, C1R, CST7, CTLA4, IGKC, IRF8, IL18R1, IL2RA, IL2RG, IL7R, LCP2, HLA-B, HLA-DMB, HLA-DOB, HLA-DQA1, HLA-DRB1, MARCH1, PDCD1LG2, SERPINB9, TLR4, VAV1
inflammatory response	28	1.2E-09	CCL13, CCL18, CCL19, CCL21, CCL22, CCR1, CXCL13, CD27, LYN, TNFRSF1B, TNFRSF9, AOAH, ADGRE5, F2R, C3AR1, CYBB, FPR3, GBP5, HAVCR2, IL1RL2, IL2RA, IL36G, LYZ, PRDX5, STAB1, THEMIS2, TLR4, TLR8
adaptive immune response	17	1.0E-08	BTK, CLEC4M, CD4, CD84, CD86, JAK3, LYN, SLAMF7, CTSH, CTSS, CTLA4, HAVCR2, LAIR1, LAMP3, PAG1, PRKCB, TAP1
T cell costimulation	12	1.4E-07	CCL19, CCL21, CD28, CD4, CD80, CD86, LYN, CTLA4, HLA-DQA1, HLA-DRB1, PDCD1LG2, VAV1
B cell receptor signaling pathway	10	4.6E-07	BTK, LYN, NCKAP1L, CTLA4, IGKC, KLHL6, NFATC2, PRKCB, PTPRC, RFTN1
positive regulation of T cell proliferation	10	1.2E-06	CCL19, CD28, CD4, JAK3, NCKAP1L, SASH3, HAVCR2, HLA-DMB, PDCD1LG2, PTPRC
innate immune response	23	1.1E-05	BTK, CLEC4M, CD84, JAK3, LYN, SLAMF6, APOL1, C1QA, C1QC, C1RL, C1R, C2, CYBB, HAVCR2, IGKC, IL36G, HLA-B, PGLYRP4, SERPING1, TLR4, TLR8, TMEM173
T cell differentiation	7	1.3E-05	CD4, IL7R, PREX1, PTPN22, PTPRC, RUNX2, VAV1
T cell receptor signaling pathway	13	1.5E-05	CD28, CD4, WAS, INPP5D, LCP2, HLA-DQA1, HLA-DRB1, PAG1, PTPN22, PTPRC, PTPRJ, RFTN1, THEMIS2
signal transduction	42	2.6E-05	BCL11A, CCL13, CCL18, CCL22, CD4, CD53, CD74, CDC42EP3, FCGR2B, GRK3, LYN, NEK6, RASA3, RASAL3, RASSF4, ARHGAP25, SH2D2A, SP110, SPOCK2, TRAF1, APBB1IP, CSF2RB, FPR3, INPP4A, INPP4B, INPP5D, IL1RL2, IL18R1, IL2RG, IL7R, LPXN, LITAF, HLA-DOB, MRC1, PITPNC1, PAG1, PRKCB, P2RX4, RAC2, STX2, TENM2, ZYX
positive regulation of neutrophil chemotaxis	6	3.7E-05	CCL19, CCL21, CD74, NCKAP1L, C3AR1, RAC2
antigen processing and presentation	8	5.5E-05	CLEC4M, CD74, CTSH, CTSS, HLA-B, HLA-DMB, HLA-DQA1, HLA-DRB1
chemotaxis	11	7.1E-05	CCL13, CCL18, CCL22, CCR1, CMTM3, CMTM7, NCKAP1L, C3AR1, DOCK2, PLD1, RAC2
positive regulation of GTPase activity	25	9.2E-05	ADAP2, CCL13, CCL18, CCL19, CCL22, CDC42EP3, JAK3, NCKAP1L, RASA3, RASAL3, ARHGEF6, RAPGEFL1, ARHGAP18, ARHGAP25, CSF2RA, CSF2RB, CYTH4, DOCK2, FGFR3, IL2RA, IL2RG, PREX1, PLEKHG6, SMAP2, VAV1

platelet activation	10	2.3E-04	GNG2, LYN, F2RL2, F2R, F5, LCP2, PLSCR1, PRKCB, RAC2, VAV1
positive regulation of ERK1 and ERK2 cascade	12	3.3E-04	CCL13, CCL18, CCL19, CCL21, CCL22, CCR1, CD74, F2R, FGFR3, HAVCR2, PTPN22, TLR4
leukocyte migration	10	3.6E-04	CD74, CD84, LYN, C3AR1, FPR3, INPP5D, ITGA4, MMP9, SELL, SELPLG
cellular response to interferon-gamma	7	5.4E-04	CCL13, CCL18, CCL19, CCL21, CCL22, GBP5, MRC1
positive regulation of B cell proliferation	6	6.3E-04	CD74, NCKAP1L, SASH3, NFATC2, PTPRC, TLR4
cell adhesion	20	6.8E-04	CCR1, CD4, SLAMF7, ADGRE5, CDSN, FEZ1, HAPLN3, ITGA4, LAMC2, LPXN, NLGN4Y, NRP2, PTPRF, SELL, SELPLG, STAB1, TNC, THEMIS2, TGFBI, ZYX
cell-cell signaling	14	7.2E-04	CCL13, CCL18, CCL21, CCL22, CCR1, CXCL13, CD80, CD86, ADGRE5, C1QA, FGFR3, IL36G, STAB1, ZYX
immunoglobulin mediated immune response	4	8.5E-04	CD27, CD74, INPP5D, TLR8
positive regulation of interleukin-2 biosynthetic process	4	1.1E-03	CD28, CD4, CD80, CD86
negative regulation of immune response	4	1.1E-03	LYN, CTLA4, INPP5D, IL2RA
defense response	7	1.3E-03	CD74, CD84, SP140, WAS, HLA-B, RNASE6, TAP1
cell surface receptor signaling pathway	14	1.4E-03	CCR1, CXCL13, CD27, CD28, CD37, CD4, CD53, ADGRE5, BIRC3, IFITM1, IL2RA, IL7R, PTPRC, TSPAN11
positive regulation of interferon-gamma production	6	1.4E-03	SASH3, SLAMF6, HAVCR2, IRF8, IL18R1, TLR4
positive regulation of protein kinase activity	6	1.5E-03	CCL19, CCL21, CD4, RASSF2, LCP2, PTPRC
lymphocyte chemotaxis	5	1.5E-03	CCL13, CCL18, CCL19, CCL21, CCL22
chemokine-mediated signaling pathway	7	1.7E-03	CCL13, CCL18, CCL19, CCL21, CCL22, CCR1, CXCL13
positive regulation of B cell differentiation	4	1.8E-03	BTK, CD27, NCKAP1L, INPP5D
regulation of actin filament polymerization	4	1.8E-03	FCHSD2, ARHGAP18, HCLS1, PREX1
positive regulation of T cell differentiation	4	2.2E-03	CD27, CD74, IL1RL2, IL2RA
viral entry into host cell	7	3.2E-03	CLEC4M, CD80, CD86, ANPEP, LAMP3, MRC1, SELPLG
antigen processing and presentation of peptide or polysaccharide antigen via MHC class II	4	3.2E-03	HLA-DMB, HLA-DOB, HLA-DQA1, HLA-DRB1
apoptotic process	21	3.3E-03	DAB2, GPR65, NEK6, PIM2, ARHGEF6, TRAF1, TNFRSF9, BIRC3, CTSH, GSDMA, INPP5D, IL2RA, LITAF, PRDX5, PLSCR1, PDCD4, PRKCB, RNF130, STK17A, SERPINB9, TMEM173
cellular response to lipopolysaccharide	8	4.3E-03	CD80, CD86, TNFRSF1B, HAVCR2, IRF8, LITAF, MRC1, TLR4
establishment of T cell polarity	3	4.6E-03	CCL19, CCL21, DOCK2
negative regulation of leukocyte apoptotic process	3	4.6E-03	CCL19, CCL21, HCLS1

synapse assembly	6	4.8E-03	RAB29, SPOCK2, CLSTN3, NLGN4Y, PCDHB16, SDK1
cell chemotaxis	6	6.2E-03	CCL13, CCL19, CCL21, BIN2, C3AR1, FPR3
release of sequestered calcium ion into cytosol	5	6.2E-03	CCL19, CCL21, RASA3, F2R, PTPRC
antigen processing and presentation of exogenous peptide antigen via MHC class II	7	6.3E-03	CD74, IFI30, CTSS, HLA-DMB, HLA-DOB, HLA-DQA1, HLA-DRB1
positive regulation of dendritic cell antigen processing and presentation	3	6.4E-03	CCL19, CCL21, CD74
positive regulation of B cell receptor signaling pathway	3	6.4E-03	CMTM3, LYN, PRKCB
neutrophil chemotaxis	6	6.7E-03	CCL13, CCL18, CCL22, NCKAP1L, PREX1, VAV1
monocyte chemotaxis	5	6.7E-03	CCL13, CCL18, CCL19, CCL21, CCL22
cellular response to cytokine stimulus	4	6.9E-03	CD86, DPYSL3, HCLS1, ITGA4
Fc-gamma receptor signaling pathway involved in phagocytosis	8	8.1E-03	FCGR2A, LYN, NCKAP1L, WAS, ARPC1B, IGKC, MYO1G, VAV1
toll-like receptor 3 signaling pathway	3	8.4E-03	CD86, HAVCR2, RFTN1
positive regulation of I-kappaB kinase/NF-kappaB signaling	9	8.8E-03	CCL19, CCL21, NEK6, PIM2, BIRC3, F2R, LITAF, PRKCB, UBD
cortical actin cytoskeleton organization	4	8.8E-03	NCKAP1L, FMNL1, FMNL2, PLEK
complement activation, classical pathway	7	8.9E-03	C1QA, C1QC, C1RL, C1R, C2, IGKC, SERPING1
positive regulation of filopodium assembly	4	1.1E-02	CCL21, DPYSL3, TENM2, TGFB3
positive regulation of Rho protein signal transduction	4	1.1E-02	GPR65, F2RL2, F2R, P2RY8
thrombin receptor signaling pathway	3	1.1E-02	F2RL2, F2R, PLEK
cell migration	9	1.3E-02	JAK3, CCDC88A, FMNL3, LCP1, NFATC2, PSTPIP2, PTPRF, SDC3, USP9Y
immunological synapse formation	3	1.3E-02	CCL19, CCL21, DOCK2
positive regulation of cytosolic calcium ion concentration involved in phospholipase C-activating G-protein coupled signaling pathway	4	1.4E-02	GPR65, F2RL2, F2R, P2RY8
negative regulation of interferon-gamma production	4	1.4E-02	HAVCR2, HLA-DRB1, PDCD1LG2, TLR4
regulation of immune response	9	1.5E-02	FCGR2B, SLAMF6, SLAMF7, IGKC, ITGA4, IFITM1, LAIR1, HLA-B, SELL
B cell activation	4	1.5E-02	BTK, CD86, IKZF3, PRKCB
negative regulation of T cell activation	3	1.6E-02	JAK3, PAG1, PTPN22
regulation of cytokine secretion	3	1.6E-02	LYN, TLR4, TLR8
negative thymic T cell selection	3	1.6E-02	CD28, CD74, DOCK2
protein complex assembly	7	1.8E-02	CD74, NCKAP1L, TAPBP, TRAF1, WAS, LPXN, PARD3
positive regulation of phosphatidylinositol 3-kinase activity	4	1.8E-02	CCL19, CCL21, LYN, FGFR3
keratan sulfate catabolic process	3	1.9E-02	FMOD, GLB1, GNS
regulation of cytokine production	3	2.2E-02	LYN, LITAF, UBASH3A
detection of bacterium	3	2.2E-02	HLA-B, HLA-DRB1, PGLYRP4
regulation of protein phosphorylation	4	2.3E-02	LYN, CCDC88A, STAT2, TLR8

negative regulation of B cell proliferation	3	2.5E-02	LYN, CTLA4, INPP5D
positive regulation of cell motility	3	2.5E-02	CCL19, CCL21, SPOCK2
response to wounding	5	2.7E-02	CCR1, F2RL2, F2R, PLLP, TNC
cell maturation	4	2.7E-02	BTK, CCL19, CCL21, RUNX2
response to lipopolysaccharide	8	2.9E-02	CXCL13, CD27, TNFRSF9, F2R, CSF2RB, MGST2, PTPN22, TLR4
positive regulation of JNK cascade	5	2.9E-02	CCL19, CCL21, CD27, RASSF2, TLR4
negative regulation of T cell proliferation	4	2.9E-02	CTLA4, HAVCR2, IL2RA, HLA-DRB1
positive regulation of transcription factor import into nucleus	3	2.9E-02	HCLS1, PTPN22, TMEM173
integrin-mediated signaling pathway	6	3.3E-02	DAB2, FERMT3, ITGA4, PLEK, VAV1, ZYX
interleukin-7-mediated signaling pathway	2	3.6E-02	IL2RG, IL7R
regulation of mast cell activation	2	3.6E-02	LYN, PLSCR1
lymphocyte aggregation	2	3.6E-02	RAC2, STK10
negative regulation of T cell receptor signaling pathway	3	3.7E-02	PTPN22, PTPRJ, UBASH3A
regulation of blood coagulation	3	3.7E-02	CLEC4M, F2R, STX2
dendritic cell chemotaxis	3	3.7E-02	CCL19, CCL21, CCR1
cellular response to interleukin-1	5	3.9E-02	CCL13, CCL18, CCL19, CCL21, CCL22
apoptotic signaling pathway	5	3.9E-02	BTK, CD28, TNFRSF1B, P2RX4, TLR4
interferon-gamma-mediated signaling pathway	5	3.9E-02	IFI30, IRF8, HLA-B, HLA-DQA1, HLA-DRB1
regulation of cell shape	7	4.1E-02	CCL13, CDC42EP3, ARHGAP18, FMNL1, FMNL3, MYH14, PTN
vascular endothelial growth factor receptor signaling pathway	5	4.1E-02	NCKAP1L, SH2D2A, CYBB, NRP2, VAV1
positive regulation of innate immune response	3	4.1E-02	GBP5, PLSCR1, TLR8
positive regulation of inflammatory response	5	4.2E-02	CCL13, CCL18, ETS1, PLA2G7, TLR4
negative regulation of neuron projection development	4	4.4E-02	BCL11A, RAB29, DPYSL3, ITM2C
positive regulation of interleukin-4 production	3	5.0E-02	CD28, SASH3, HAVCR2
