# Supplementary material for: Gene signatures associated with barrier dysfunction and infection in oral lichen planus identified by analysis of transcriptomic data
Source: PLoS One. 2021 Sep 10;16(9):e0257356. doi: 10.1371/journal.pone.0257356 (PMC8432868; doi:10.1371/journal.pone.0257356)
Supplement: S9 Table — (PDF) [file pone.0257356.s009.pdf]

Upregulated, Downregulated

S9 Table. Gene Ontology biological process terms enriched in the common DEGs of the epithelium and mucosa partial datasets

Term	Count	p-value	Gene
negative regulation of cell adhesion	3	3.0E-03	LPXN, TNC, TGFBI
oxidation-reduction process	6	9.2E-03	ALDH3A1, CBR1, CYP11A1, CYP4F12, MAOA, PGD
transmembrane receptor protein tyrosine phosphatase signaling pathway	2	1.3E-02	PTN, PTPRF
positive regulation of neuron projection development	3	1.6E-02	BCL11A, FEZ1, PTN
cellular response to vitamin D	2	1.7E-02	PTN, TNC
cell adhesion	5	1.8E-02	FEZ1, LPXN, PTPRF, TNC, TGFBI
positive regulation of cell proliferation	5	1.9E-02	ALDH3A1, CCND2, FGFR3, PTN, TNC
